# Supplementary material for: B1a and B2 cells are characterized by distinct CpG modification states at DNMT3A-maintained enhancers
Source: Nat Commun. 2021 Apr 13;12:2208. doi: 10.1038/s41467-021-22458-9 (PMC8044213; doi:10.1038/s41467-021-22458-9)
Supplement: Supplementary file 1 — Supplementary Information [file 41467_2021_22458_MOESM1_ESM.pdf]

SUPPLEMENTARY INFORMATION

B1a and B2 cells are characterized by distinct CpG modification states at DNMT3A-maintained enhancers

Vinay S. Mahajan*[1,2], Hamid Mattoo* [1,3], Na Sun* [1,4], Vinayak Viswanadham* [1,5], Grace J. Yuen[1], Hugues Allard-Chamard [1], Maimuna Ahmad [1], Samuel JH Murphy [1], Annaiah Cariappa [1], Yesim Tuncay [1] and Shiv Pillai [1]

[1] Ragon Institute of MGH, MIT and Harvard, Cambridge, MA, USA

[2] Brigham and Women's Hospital, Boston, MA, USA

[3] Immunology and Inflammation Therapeutic Area, Sanofi, Cambridge, MA, USA

[4] Department of Electrical Engineering and Computer Science, MIT, Cambridge, MA, USA

[5] Department of Biomedical Informatics, Harvard Medical School, Boston, MA, USA

*These authors contributed equally

Corresponding author and lead contact: pillai@helix.mgh.harvard.edu

Supplementary Figure 1: Overlap between genes upregulated during differentiation (from proB2 to B2) and genes in the proximity of hypomethylated DMRs in B2 cells (p = 2.52e-7, two-sided Fisher's exact test).

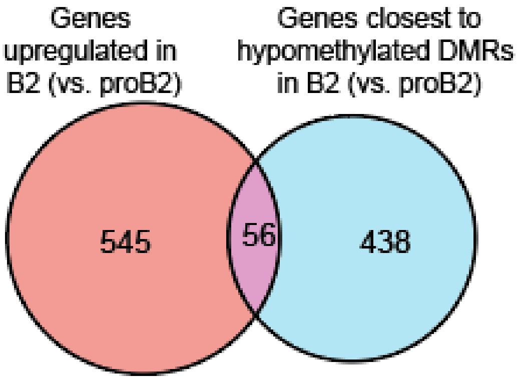

Supplementary Figure 2: Association between the number of DMRs and changes in gene expression during B1 development

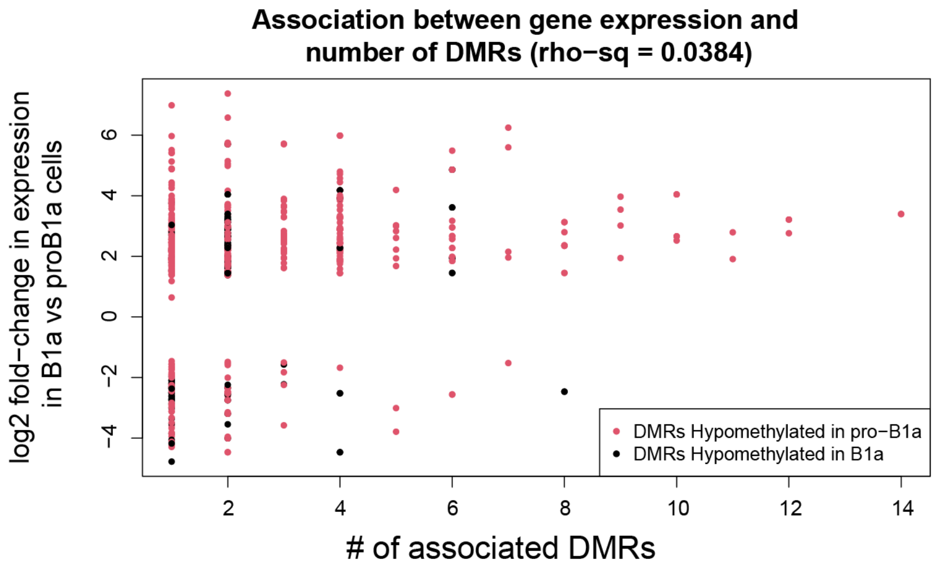

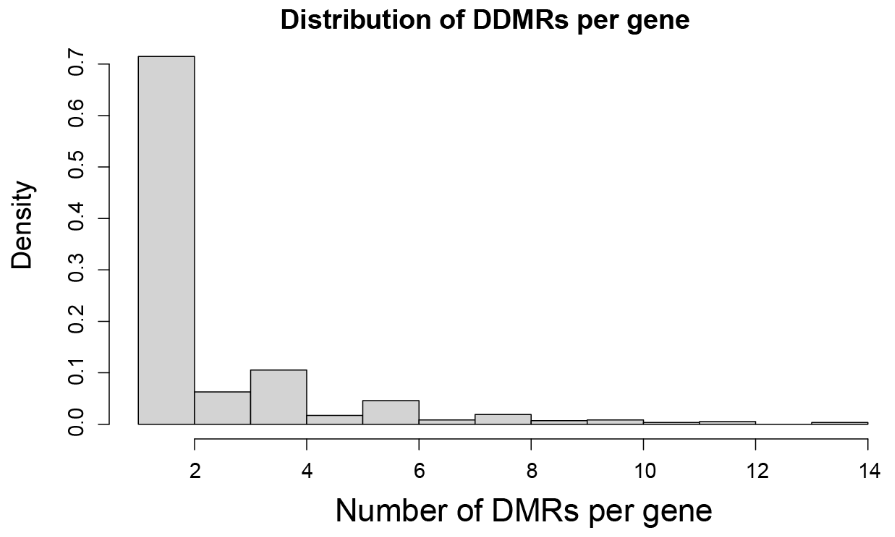

Supplementary Figure 3: B1a and B2 cell progenitors and marginal zone B cells in *Dnmt3a*$^{-/-}$ mice

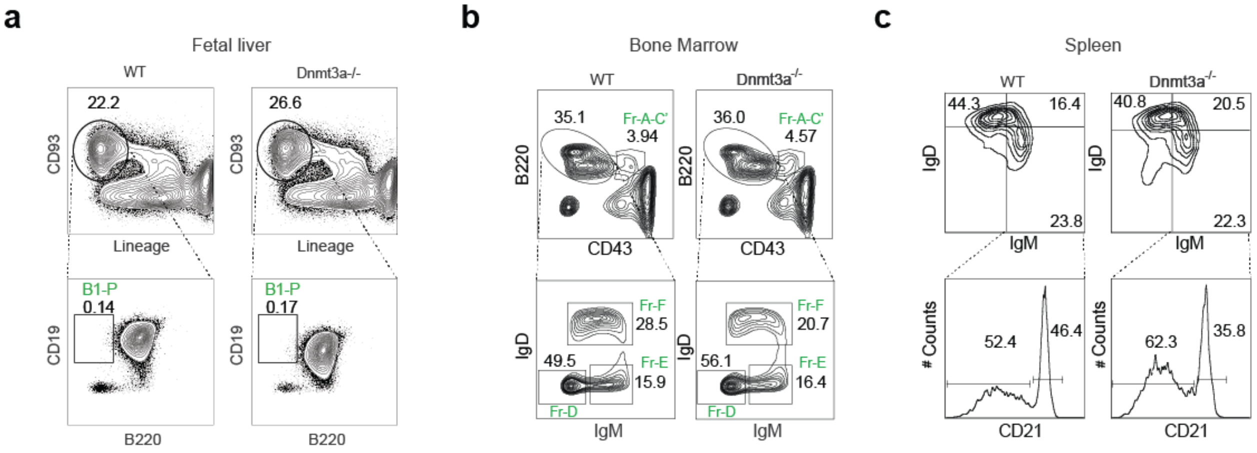

(a) Frequency of fetal liver B1 progenitors (B1-P) in wild-type and *Dnmt3a*$^{-/-}$ E16 mouse fetuses (gated as lineage-negative CD93$^+$ CD19$^+$ B220$^-$).
(b) Frequency of developing (Fractions A-E) B2 cells and recirculating (Fr-F) mature B2 subsets in the bone marrow of 4 week old wild-type and *Dnmt3a*$^{-/-}$ mice.
(c) Frequency of follicular (CD19$^+$IgD$^+$IgM$^-$) and marginal zone B cells (CD19$^+$IgD$^-$ IgM$^+$CD21$^+$) in the spleens of 4 week old wild-type and *Dnmt3a*$^{-/-}$ mice.

Supplementary Figure 4: IgH and IgL repertoire in wild-type and *Dnmt3a*$^{-/-}$ B1a and B2 cells.

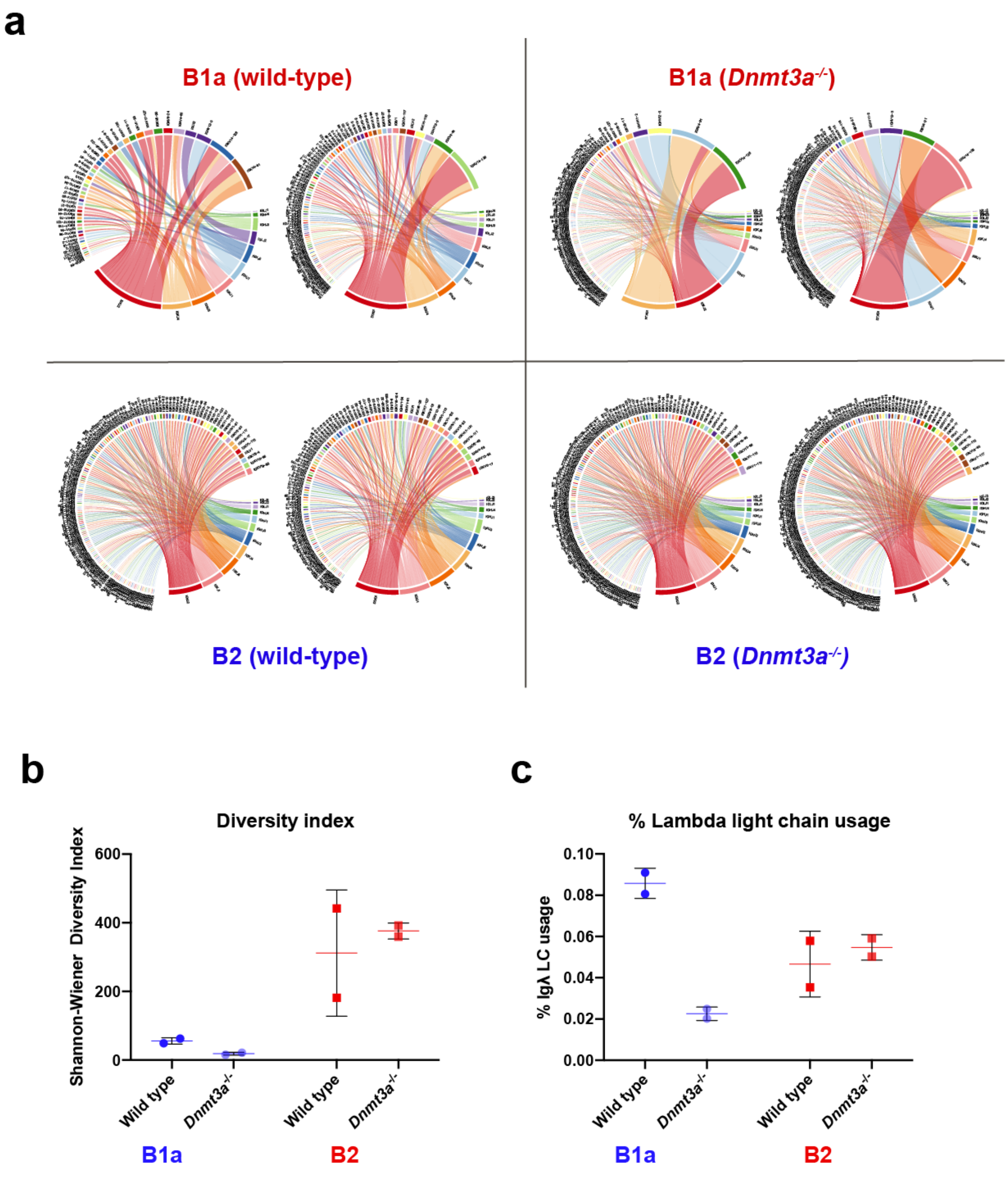

(a) The IgH and IgL repertoire of B1a and B2 cells from 4-week-old mice plotted as circos plots based on unique VJ pairing. IgH and IgL transcripts were extracted from RNAseq reads (n = two animals each). The corresponding estimates of **(b)** the CDR3 clonal diversity in terms of the Shannon-Wiener index and **(c)** the lambda light chain usage are depicted in barcharts (n = 2, mean + SD).

Supplementary Figure 5:

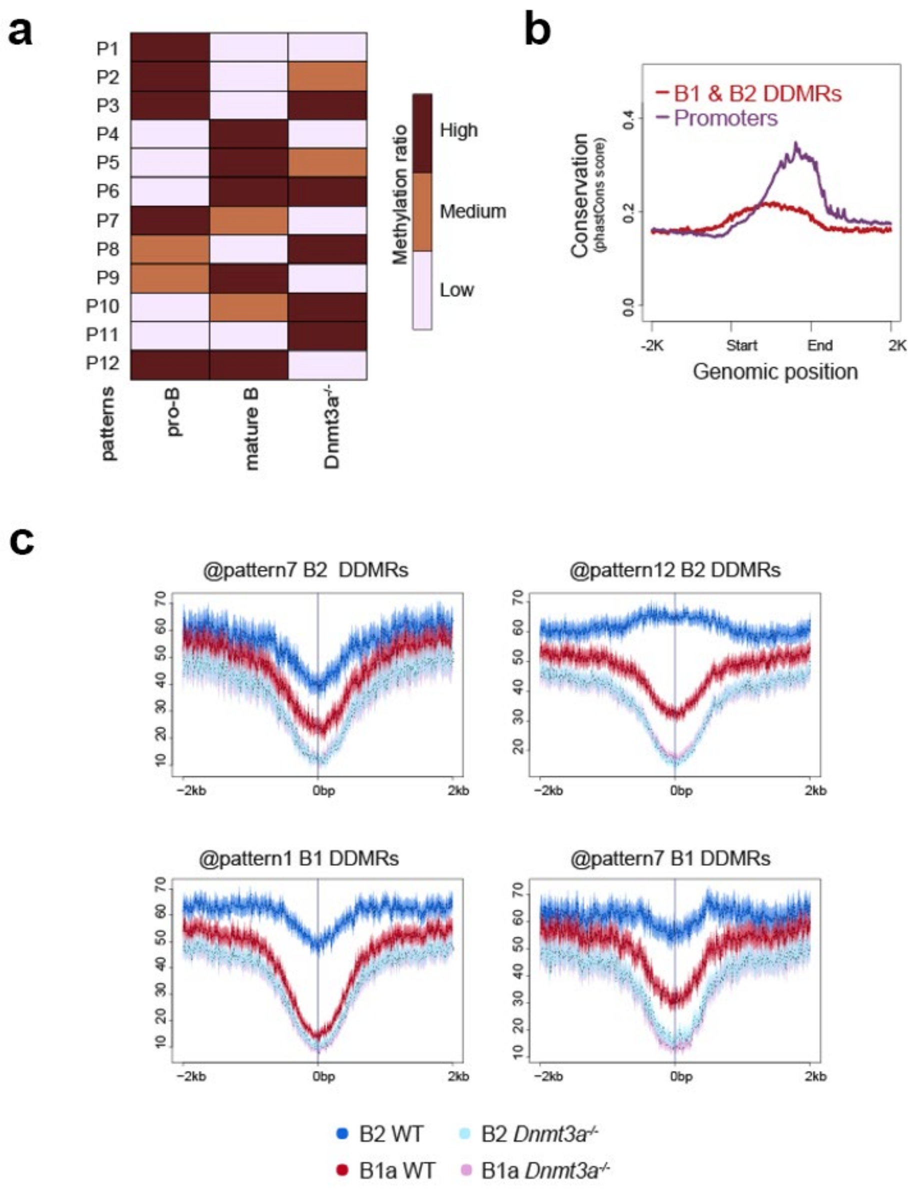

(a) Schematic of 12 possible patterns of average CpG methylation level in DDMRs across 3 cell types (proB, wild-type mature B, and *Dnmt3a*$^{-/-}$ mature B cells). Each B1 and B2 DDMR was subsequently assigned to its best matched pattern.
(b) CpG modification profiles measured by WGBS plotted +/- 2kb from the center of the B2 DDMRs (patterns 7 and 12) and B1 DDMRs (pattern 1 and 7) in B1 and B2 cells.
(c) DNA sequence conservation across 60 vertebrate species in B lineage DDMRs. All promoter intervals are shown for comparison. The phastCons scores for multiple alignments of 59 vertebrate genomes to the mouse genome from the UCSC phastCons60way track were used.

Supplementary Figure 6: Distribution of distances between B1 or B2 DDMRs and the nearest TSS. The six most abundant DDMR patterns are depicted.

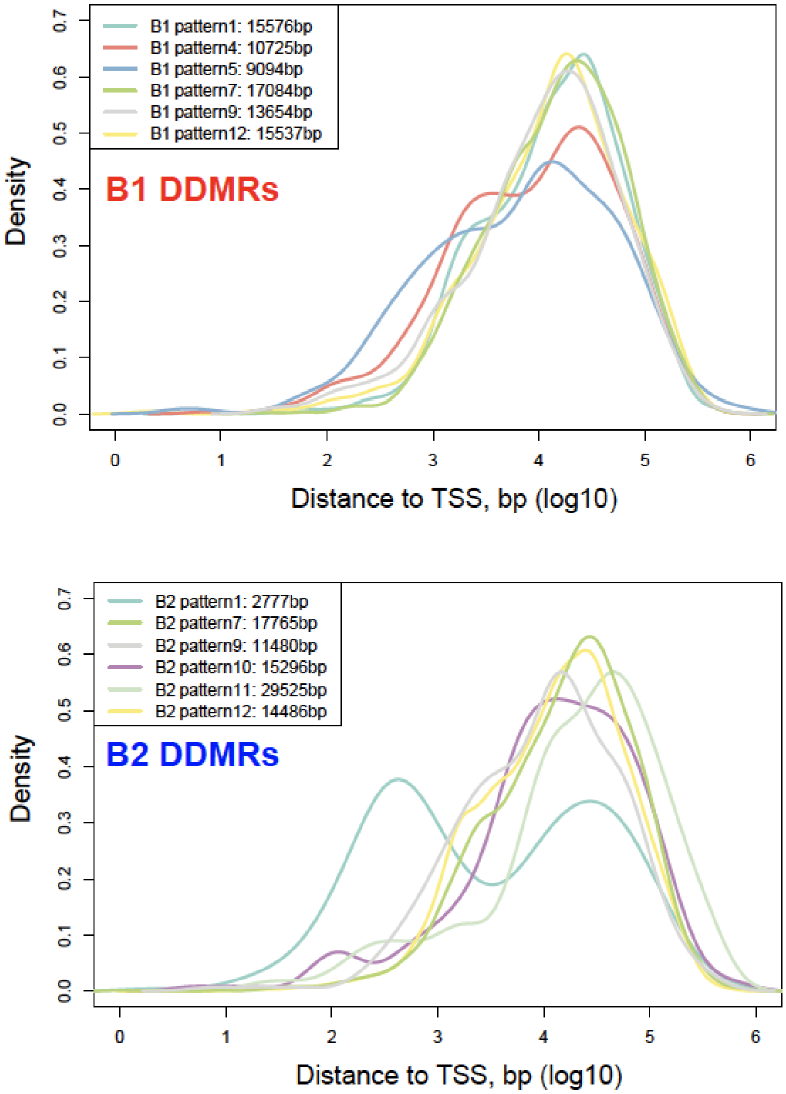

Supplementary Figure 7: ChromHMM profiles in B1 and B2 DDMRs

Enrichment of publicly available ChromHMM profiles calculated based on mouse ENCODE data in CH12 cells and mouse spleen. CH12 cells are a mouse-derived CLL-like B-cell lymphoma with a B1a surface marker expression profile ($IgM^{+}CD5^{+}CD23^{-}$).

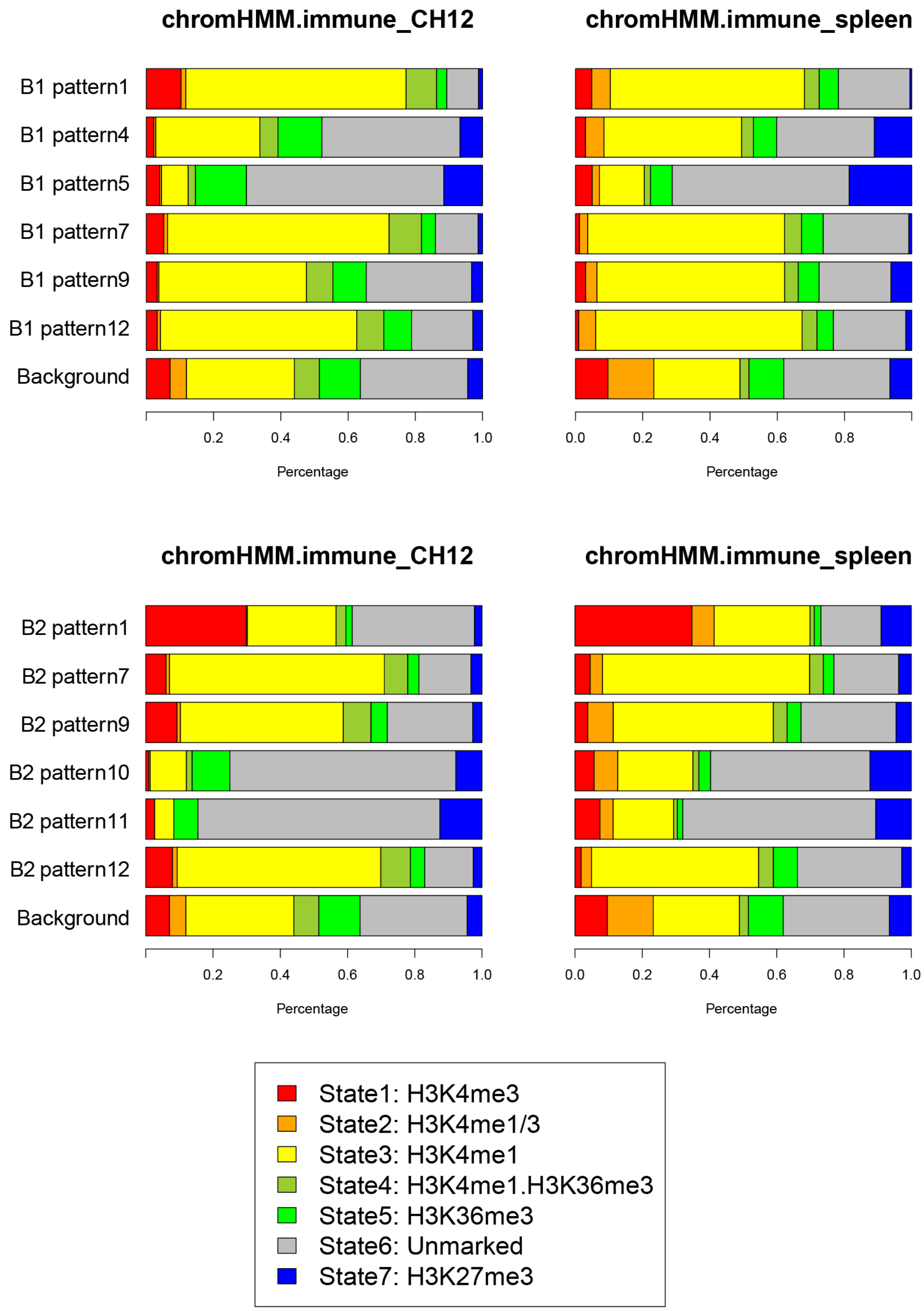

Supplementary Figure 8: DNMT3A-maintained enhancers

A schematic illustration of the presumed dynamic interplay between TET2 and DNMT3A at DMEs in B1a and B2 cells and how it differs between the two B cell lineages.
(this schematic was created by the authors using BioRender.com)

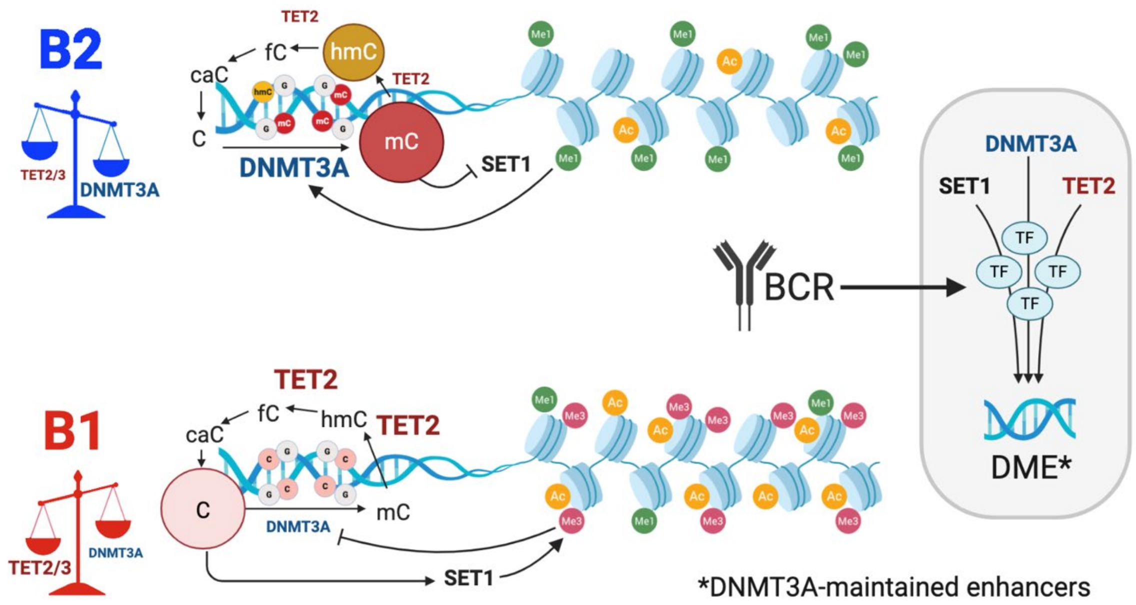

Supplementary Figure 9: Flow cytometry gating strategy used for identifying B1a, B2, proB1 and proB2 cells.

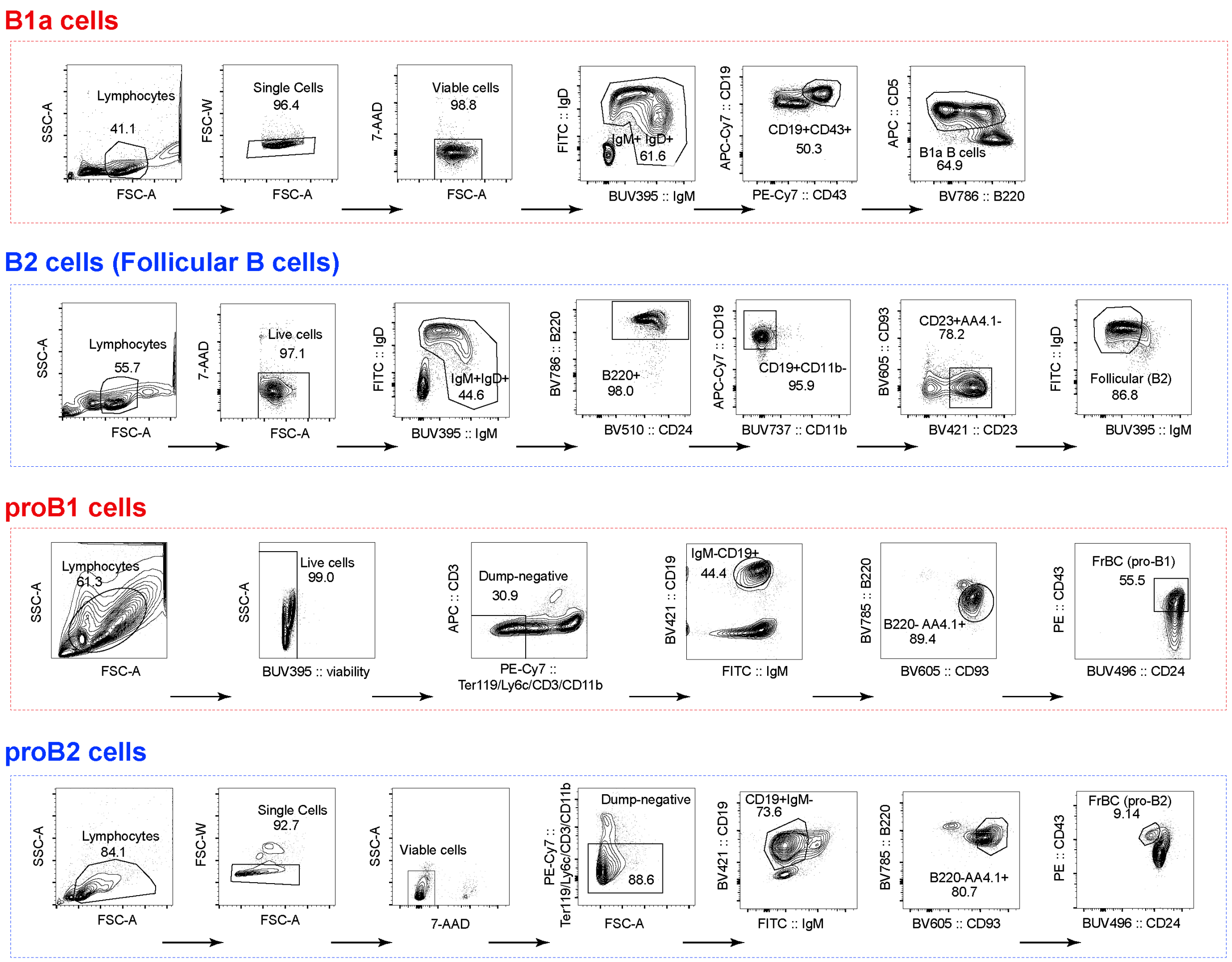

Supplementary Table 1: GO terms (KEGG pathways) enriched in the vicinity of enhancers that are differentially methylated during B1a cell development.

**GO term enrichment (KEGG pathways)**			
**Genes in the vicinity of enhancers hypomethylated in B1a cells (n=2048)**			
Enrichment FDR	*Genes in list*	*Total genes*	*Functional Category*
2.60E-16	35	70	B cell receptor signaling pathway
4.00E-16	113	532	Pathways in cancer
6.20E-14	39	101	T cell receptor signaling pathway
1.80E-12	37	101	Th17 cell differentiation
3.80E-12	48	163	Hepatitis B
**Genes in the vicinity of enhancers hypermethylated in B1a cells (n=830)**			
Enrichment FDR	*Genes in list*	*Total genes*	*Functional Category*
6.20E-07	49	532	Pathways in cancer
7.10E-05	16	100	Melanogenesis
7.90E-05	20	159	Wnt signaling pathway
7.90E-05	20	159	Cushing syndrome
1.40E-04	19	153	Hippo signaling pathway

Supplementary Table 2: Summary of WGBS and TAB-Seq data.

	Sample	Aligned dedup Reads	C to T conversion rate	Estimated coverage (symmetric CpG)	Estimated coverage (all)	Percentage of modified C in three contexts		
						CpG	CHG	CHH
WGBS	proB1	144,594,101	0.9924	5.39	3.18	74.30%	0.60%	0.80%
	B1a wild type	204,542,891	0.9858	6.69	3.57	73.10%	1.20%	1.50%
	B1a Dnmt3a-/-	197,693,927	0.9893	7.23	3.79	69.10%	0.70%	1.10%
	proB2	215,500,341	0.9922	7.59	4.2	76.00%	0.60%	0.80%
	B2 wild type	197,191,769	0.9885	7.22	3.94	76.00%	1.00%	1.20%
	B2 Dnmt3a-/-	151,323,952	0.9917	5.34	3.04	71.10%	0.70%	0.90%
TAB-Seq	B1a wild type	186,911,414	0.9871	7.08	2.73	2.60%	1.10%	1.30%
	B2 wild type	169,148,197	0.9860	6.22	2.54	3.80%	1.20%	1.40%
