# Supplementary material for: B1a and B2 cells are characterized by distinct CpG modification states at DNMT3A-maintained enhancers
Source: Nat Commun. 2021 Apr 13;12:2208. doi: 10.1038/s41467-021-22458-9 (PMC8044213; doi:10.1038/s41467-021-22458-9)
Supplement: Supplementary file 2 — Reporting Summary [file 41467_2021_22458_MOESM2_ESM.pdf]

nature research

Corresponding author(s): Shiv Pillai

Last updated by author(s): 2/24/2021

Reporting Summary

Nature Research wishes to improve the reproducibility of the work that we publish. This form provides structure for consistency and transparency in reporting. For further information on Nature Research policies, see Authors & Referees and the Editorial Policy Checklist .

Statistics

For all statistical analyses, confirm that the following items are present in the figure legend, table legend, main text, or Methods section.

n/a	Confirmed	
☐	☒	The exact sample size (n) for each experimental group/condition, given as a discrete number and unit of measurement
☒	☐	A statement on whether measurements were taken from distinct samples or whether the same sample was measured repeatedly
☐	☒	The statistical test(s) used AND whether they are one- or two-sided<br>*Only common tests should be described solely by name; describe more complex techniques in the Methods section.*
☒	☐	A description of all covariates tested
☐	☒	A description of any assumptions or corrections, such as tests of normality and adjustment for multiple comparisons
☐	☒	A full description of the statistical parameters including central tendency (e.g. means) or other basic estimates (e.g. regression coefficient) AND variation (e.g. standard deviation) or associated estimates of uncertainty (e.g. confidence intervals)
☐	☒	For null hypothesis testing, the test statistic (e.g. F, t, r) with confidence intervals, effect sizes, degrees of freedom and P value noted<br>*Give P values as exact values whenever suitable.*
☒	☐	For Bayesian analysis, information on the choice of priors and Markov chain Monte Carlo settings
☒	☐	For hierarchical and complex designs, identification of the appropriate level for tests and full reporting of outcomes
☐	☒	Estimates of effect sizes (e.g. Cohen's d, Pearson's r), indicating how they were calculated

Our web collection on statistics for biologists contains articles on many of the points above.

Software and code

Policy information about availability of computer code

Data collection	FACS Diva v9
Data analysis	FlowJo v10<br>bowtie/1.2.1.1<br>bcl2fastq/2.18.0.12<br>cutadapt/1.11<br>bedtools/2.27.1<br>rsem/1.25.0<br>homer/4.10.3<br>methpipe/3.4.3<br>bismark/0.18.1

For manuscripts utilizing custom algorithms or software that are central to the research but not yet described in published literature, software must be made available to editors/reviewers. We strongly encourage code deposition in a community repository (e.g. GitHub). See the Nature Research guidelines for submitting code & software for further information.

Data

Policy information about availability of data

All manuscripts must include a data availability statement. This statement should provide the following information, where applicable:

- Accession codes, unique identifiers, or web links for publicly available datasets
- A list of figures that have associated raw data
- A description of any restrictions on data availability

Data is publicly available at: https://www.ncbi.nlm.nih.gov/geo/query/acc.cgi?acc=GSE150497
GEO accession: GSE150497

Field-specific reporting

Please select the one below that is the best fit for your research. If you are not sure, read the appropriate sections before making your selection.

☒ Life sciences ☐ Behavioural & social sciences ☐ Ecological, evolutionary & environmental sciences

For a reference copy of the document with all sections, see nature.com/documents/nr-reporting-summary-flat.pdf

Life sciences study design

All studies must disclose on these points even when the disclosure is negative.

Sample size	We aimed for 3 replicates where possible given sequencing bandwidth limitations. No sample size estimate was used as this was an exploratory analysis.
Data exclusions	No data were excluded from the manuscript.
Replication	The number of replicates are indicated in the figures and supplementary tables.
Randomization	No randomization was performed. Age and sex-matched mice were used in the comparison groups (wild-type vs Dnmt3a-knockout).
Blinding	Not relevant to the study. The genotypes of the animals were determined by the investigators prior to the analysis.

Reporting for specific materials, systems and methods

We require information from authors about some types of materials, experimental systems and methods used in many studies. Here, indicate whether each material, system or method listed is relevant to your study. If you are not sure if a list item applies to your research, read the appropriate section before selecting a response.

Materials & experimental systems

n/a	Involved in the study	
☐	☒	Antibodies
☒	☐	Eukaryotic cell lines
☒	☐	Palaeontology
☐	☒	Animals and other organisms
☒	☐	Human research participants
☒	☐	Clinical data

Methods

n/a	Involved in the study	
☒	☐	ChIP-seq
☐	☒	Flow cytometry
☒	☐	MRI-based neuroimaging

Antibodies

Antibodies used

#Fluorochrome Antigen (Clone) Supplier Catalog_number
APC/Cy7 anti-mouse CD19 (Clone 6D5), Biolegend #115530
PerCP/Cy5.5 anti-mouse CD45R (Clone HI100), Biolegend #304122
APC anti-mouse IgM (Clone RMM-1), Biolegend #406516
FITC anti-mouse IgD (Clone 11-26c.2a), Biolegend #405703
PE/Cy7 anti-mouse CD93 (Clone AA4.1), Biolegend #136505
PE anti-mouse CD21 (Clone 7E9), Biolegend #123410
Pacific Blue anti-mouse CD23 (Clone B3B4), Biolegend #101615
FITC anti-mouse CD3 (Clone 17A2), Biolegend #100204
PE anti-mouse IgM (Clone RMM-1), Biolegend #406507
Pacific Blue anti-mouse CD11b (Clone M1/70), Biolegend #101223

APC anti-mouse CD5 (Clone 53-7.3), Biolegend #100626
PE/Cy7 anti-mouse CD23 (Clone B3B4), Biolegend #101613
PE anti-mouse CD43 (Clone S11), Biolegend #143205
Alexa Fluor488 anti-mouse CD24 (Clone M1/69), Biolegend #101815
Brilliant Violet 421 anti-mouse CD19 (Clone 6D5), Biolegend #115537
BV786 anti-mouse B220 (Clone RA3-6B2), Biolegend #103246
BUV395 anti-mouse IgM (Clone R6-60.2), BD Biosciences #564025
BV510 anti-mouse CD24 (Clone M1/69), Biolegend #101831
BUV737 anti-mouse CD11b (Clone M1/70), BD Biosciences #564443
BV421 anti-mouse CD23 (Clone B3B4), Biolegend #101621
BV605 anti-mouse CD93 (Clone AA4.1), BD Biosciences #740386
BUV496 anti-mouse CD24 (Clone B3B4), BD Biosciences #564664

Validation

Validation data supplied by the manufacturer was used as a guideline, and additional validation and antibody titration was performed using positive/negative control cells from C57BL/6 mice prior to the experiments.

Animals and other organisms

Policy information about studies involving animals; ARRIVE guidelines recommended for reporting animal research

Laboratory animals

C57BL/6 mice and CD19-Cre (Jax Stock No: 006785) and Dnmt3a-tm3.1Enl (Kaneda et al.): Mice of both sexes and less than 9 months old were used in this study.

Wild animals

No wild animals were used in this study

Field-collected samples

No field-collected samples were used in this study.

Ethics oversight

Institutional Animal Care and Use Committee (IACUC)

Note that full information on the approval of the study protocol must also be provided in the manuscript.

Flow Cytometry

Plots

Confirm that:

- [x] The axis labels state the marker and fluorochrome used (e.g. CD4-FITC).
- [x] The axis scales are clearly visible. Include numbers along axes only for bottom left plot of group (a 'group' is an analysis of identical markers).
- [x] All plots are contour plots with outliers or pseudocolor plots.
- [x] A numerical value for number of cells or percentage (with statistics) is provided.

Methodology

Sample preparation

Single cell suspensions were made from spleens, bone marrow, peritoneal washes or fetal liver of Dnmt3a-/- or WT mice with gentle disaggregation followed by red blood cell
lysis using ACK buffer (Lonza). They were stained with labeled antibodies and analyzed by flow cytometry.

Instrument

BD LSR2 or BD FACS Aria

Software

FlowJo or FACS Diva

Cell population abundance

>99%

Gating strategy

Included in the methods and supplementary information

- [x] Tick this box to confirm that a figure exemplifying the gating strategy is provided in the Supplementary Information.
